# Selection and classification of gene expression in autism disorder: Use of a combination of statistical filters and a GBPSO-SVM algorithm

**DOI:** 10.1371/journal.pone.0187371

**Published:** 2017-11-02

**Authors:** Shilan S. Hameed, Rohayanti Hassan, Fahmi F. Muhammad

**Affiliations:** 1 Department of Computer Science, Faculty of Computing, Universiti Teknologi Malaysia, Johor Bahru, Malaysia; 2 Department of Software and Informatics Engineering, College of Engineering, Salahaddin University, Erbil, Kurdistan Region, Iraq; 3 Department of Software Engineering, Faculty of Computing, Universiti Teknologi Malaysia, Johor Bahru, Malaysia; 4 Department of Physics, Faculty of Science & Health, Koya University, Koya, Kurdistan Region, Iraq; Harbin Institute of Technology Shenzhen Graduate School, CHINA

## Abstract

In this work, gene expression in autism spectrum disorder (ASD) is analyzed with the goal of selecting the most attributed genes and performing classification. The objective was achieved by utilizing a combination of various statistical filters and a wrapper-based geometric binary particle swarm optimization-support vector machine (GBPSO-SVM) algorithm. The utilization of different filters was accentuated by incorporating a mean and median ratio criterion to remove very similar genes. The results showed that the most discriminative genes that were identified in the first and last selection steps included the presence of a repetitive gene (CAPS2), which was assigned as the gene most highly related to ASD risk. The merged gene subset that was selected by the GBPSO-SVM algorithm was able to enhance the classification accuracy.

## Introduction

Autism spectrum disorder (ASD) is a neurodevelopmental disorder that is defined by weakened social interactions, impaired verbal and non-verbal communication and repetitive actions [1, 2]. ASD affects more than 1% of the population, and males are four times more vulnerable to the disorder than females [3]. Although environmental factors are believed to contribute to autism, researchers believe that genetic factors play a major role in the occurrence of the disorder [4]. In a study of twins, the presence of high similarity in the features of autistic twins was noticed [5]. It was observed that the genetic similarity among identical twins who are from the same developmental environment and have the same parental chromosomes is high. In these contexts, biologists have attempted to identify the most relevant genes that can be utilized as biomarkers for tracing the disorder. The attribution of a role of specific genes in the development of autism enables us to understand the mechanism of development of the disorder and hence predict its serious consequences. To date, there is a lack of treatment for the major symptoms of autism, and no accurate biomarkers have been identified because the etiology of autism is not clearly known [6]. Although approximately 70% to 90% of cases of autism are thought to be related to heritable causes, the variable phenotype of the disease and the complex architecture of its genetics have made it difficult to identify specific genes that are associated with susceptibility to autism [7]. It has been claimed that the aggregate action of multiple genes is necessary to produce autism disorder, a feature that adds complexity to genomic investigations [8]. The pioneer work of Gregg *et al*. [9], which was based upon genomic profiling of whole blood, revealed differences in gene expression in autistic and healthy children. Moreover, these authors observed variations in gene expression at the early onset stage of the disease in individuals with different subtypes of autism such as autism with regression and autism without regression. Because of these variations, the identification of genes related to autism presents a difficult problem. It is quite reasonable to use gene expression data to relate the phenotypes of diseases to their attributed biomarkers [10].

Computer models can be used to study autism through the use of microarray gene expression data. A microarray is a tool that is used to estimate whether mutations in specific genes are present in a particular individual. The most common type of microarray is utilized to measure gene expression; in this type of microarray, the expression values of thousands of genes are calculated from the microarray sample [11]. Along this line, the techniques of machine learning and data mining are considered effective tools in the application of genomic medicine, which uses computational methods and genomic datasets to predict phenotypes [12]. Machine learning is valuable in the interpretation of large datasets of genomic data, and it has also been successfully utilized to annotate the wide diversity of elements in genomic sequences [13]. Genome sequence analysis has also received considerable attention. In recent years, very useful computational tools were proposed in an open-source Python package designed to formulate comprehensive built-in and user-defined features for DNA, RNA and protein sequences; these are known as representations of DNA (repDNA) [14], repRNA [15] and Pse-in-One [16], respectively. The repDNA tool was used to develop powerful computational predictors for use in identifying the biological features or attributes of DNAs by generating widely used features that reflect the physicochemical properties and sequence-order effects of DNAs and nucleotides [14]. This model includes three groups of features that can be used for different analysis purposes. In regard to RNA analysis, a new repRNA was developed to meet the increasing demands and to speed up the genome analyses [15]. The features of this model can be represented by 11 different modes of feature vectors, thereby exceeding the limitations of existing machine-learning methods such as SVM and KNN that use only vectors and not sequences. Pse-in-one [16] was proposed as an effective tool that can handle the analysis of more than one type of sample; however, it is utilization is maximized to work on DNA, RNA and protein as well. The feature vectors of Pse-in-one can be easily combined with machine-learning algorithms for use in developing computational predictors and analysis methods for various tasks in bioinformatics and systems biology. Furthermore, studies in the field of cancer informatics have shown an interesting contribution of data mining and machine learning to finding related genes [17–19]. However, gene expression in autism displays some specific characteristics that make gene selection, model creation and prediction more challenging than gene expression analysis of cancers.

The major problem in the gene expression analysis of ASD is the difficulty in selection and identification of the genes that are most relevant to autism. This problem exists because the gene expression levels in autism disorder show considerable fluctuation among individuals and because the sequences of several of these genes are highly variable [20]. In general, noise in gene expression level data usually occurs due to variations associated with the experiments or the existence of alterations in the genes [21, 22]. In the case of autism, the extra variance may be linked to the presence of alterations in many genes. Another reason for this difficulty is the limited number of observations (in the range of hundreds) that have been made in comparison to the very large number of genes (in the range of tens of thousands). In machine learning, this feature is known as high dimensionality, and sophisticated methods are required to handle it. High dimensionality also exists in genome sequence analysis data, where it poses computational challenges despite the important contribution of high-throughput sequencing technology, which greatly increases the amount of available data for discriminative motif discovery (DMD) [23, 24]. DMD methods usually have to sacrifice accuracy and may fail to fully leverage the potential of large datasets. Hence, researchers have proposed the large margin motif optimizer (LMMO) [23] for refining regulatory motifs and a novel approach referred to as discriminative motif learning via AUC (DiscMLA) to identify motifs in high-throughput datasets [24]. To further reduce computational time, some researchers have combined the proposed models with various techniques for improving the scalability of large-margin type algorithms and to accelerate DiscMLA.

The foregoing shows that it is not an easy or a straightforward task to find the attributed genes of autism unless a careful analysis and investigation is made of the microarray dataset. Gene selection methods are classified into two main types: filter-based methods and wrapper-based methods [21, 25]. Because filter-based methods usually work without using a classifier, they are efficient with respect to computational time. They are preferable for use in analyzing the high-dimensional data of microarray datasets [26]. The drawback of filter-based methods is that the selected features may not have relationships to each other, and the appearance of redundant features is possible. This may decrease the accuracy of the classifier when the gene selection results are directly applied to the learning algorithm [21]. For this reason, the best choice is to use filters in the first selection process and to apply another feature selection approach that depends on classifier accuracy to choose the attributed genes in later steps. Wrappers tend to perform better in selecting discriminative genes since they take the model hypothesis into account by training and testing in the gene space [21]. When dealing with high-dimensional data such as microarray datasets, wrapper-based methods tend to be the worst choice if applied to the data directly without any preprocessing because this leads to overfitting [27]. This is because the wrapper acts by searching and comparing the performance of each gene subset with the classification algorithm prior to estimating the best subset of genes [28]. However, if wrappers are used after the application of filter methods, they require less computational time and hence work more efficiently [29]. Conventional wrappers use search algorithms to find subsets of genes through adding or removing the best features to the space based on the fitness criteria [30]. Hence, the problems of large-scale feature selection are not efficiently solved by using conventional optimization algorithms [31]. Therefore, to address the feature selection problems effectively, meta-heuristic algorithms are being adopted. There are various meta-heuristic algorithms that can be used to address feature selection issues; these include the genetic algorithm (GA) [32], ant colony optimization [33], simulated annealing [34], and particle swarm optimization (PSO) [35]. PSO and GA are two common evolutionary algorithms that are usually applied in the form of wrapper methods [31, 36]. Comparably, PSO is efficient and simple; only a few parameters are required to perform its adjustment, and hence it is a memory-enabled algorithm. Binary PSO is a modified version of the standard PSO introduced by Kennedy and Eberhart [37] to handle variables with discrete design. BPSO was shown to outperform GA when used for feature selection using the same fitness function [38]. In another study [39], BPSO was used in feature selection such that the fitness function was designed based on the rough set. BPSO was also applied to various optimization problems [17, 40, 41]. In addition, a new discrete form of the PSO, the DPSO algorithm, which is based on the particle’s best position (pbDPSO) and global best position (gbDPSO), was adopted to find the global optimal solution for a high-dimensional grid system; in this way, a reduction in the minimum computation time and an energy improvement of up to 28% were achieved [42]. Recently, a new modified version of PSO known as geometric PSO (GPSO) was proposed by Moraglio *et al*. [43] and utilized for gene selection in cancer classification by Alba *et al*. [36]. In the current work, a combination of statistical filters and wrapper algorithms incorporating GBPSO is employed for gene selection and classification in autism disorder. This is achieved through the application of various filters in parallel with a GBPSO wrapper and a support vector machine (SVM) classifier (GBPSO-SVM algorithm). Prior to the selection process, specific pre-processing operations are performed on the dataset in a creative way to remove the most similar genes. The presented results were found to improve the accuracy of gene classification in autism disorder.

## Materials and methods

### Experimental overview

The experimental procedure of the current work was implemented in three basic steps; these are briefly described below, and the details of each step are given in the following subsections.

First step: in this stage, the whole dataset was checked for the similarity of gene expression in the control and autism classes. Genes with mean or median ratios close to unity (equal to or greater than 0.95) were removed; in this way, the number of genes in the dataset was reduced from 54,613 to 9454.

Second step: in this stage, the reduced dataset was divided into two parts, 85% of which was used in the process of model training and validation (testing); the 15% non-involved set was set aside to be used as a new real-world dataset against the gene classification based on the pre-defined model. Later on, three filters, namely, the t-test (TT), feature correlation (COR) and the Wilcoxon Rank Sum test (WRS), were initially applied in parallel to select the 200 most discriminative genes using a 10-fold run evaluation.

**Fig 1:**
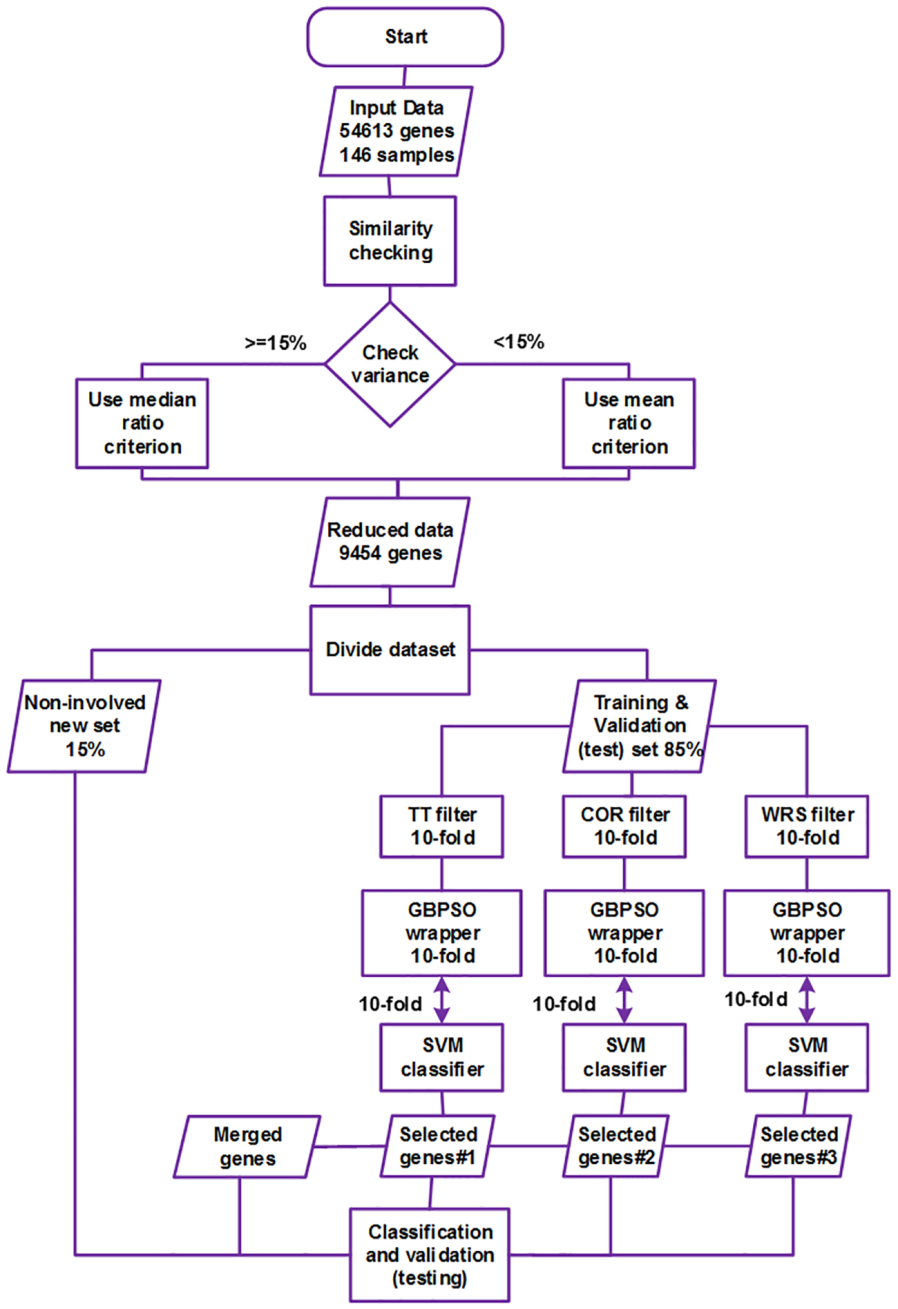
Experimental setup used to select autism-related genes and to perform classification.

Third step: in this stage, the final most discriminative subsets of genes were selected by the GBPSO-SVM algorithm, and classification was performed based on the resulting genes. Furthermore, a merged set of genes was generated from the combination of these three final subsets based on their frequencies of appearance in the 10-fold selection process. Consequently, the selected genes were used in training and validation performed with the SVM classifier in the 10-fold cross-validation scheme. Finally, the non-involved dataset mentioned in step two was used as a new real-world dataset to further test and generalize the applied model. The complete methodology of the current work is illustrated in Fig 1, and the implementation steps of the codes are given in (https://github.com/fahmi982/Implementation-Steps).

### Autism dataset

The experimental data used in the analysis comprised an autism microarray dataset that was downloaded from the well-known public repository GEO (NCBI) [44]. The dataset consists of 146 observations (samples) and 54,613 genes (features). The observations are divided into two classes, a control class containing 69 observations and an autism class containing 77 observations. Samples from autistic and control individuals were collected from persons in the Phoenix area. Blood drawing for the observations was conducted in the spring and summer of 2004. Total RNA was extracted for microarray experiments, which were performed using Affymetrix Human U133 Plus 2.0 39 Expression Arrays. The autistic patients who provided the samples were diagnosed by medical professionals (developmental pediatricians and psychologists) according to the DSM-IV criteria, and the diagnosis was confirmed on the basis of the ADOS and ADI-R criteria [45]. Samples from individuals with non-classic higher functioning forms of autism, regression and Asperger’s syndrome were not included in the dataset. Each sample was subjected to normal high-resolution chromosome analysis and had a negative result on the Fragile X DNA test.

### Pre-selection operations

High variance is one of the most apparent problems in the autism gene expression dataset used in this study; the high variance may be due to the nature of the data [46]. Moreover, the fact that the high-dimensional data in this set consisted of 54,613 genes and only 146 samples emphasizes the existence of similar expression of autism-related and non-autism-related genes. Statistical theories [47] indicate that the genes that show similar expression in both sets of individuals are not useful and that they do not include the discriminant genes. For this reason, removing very similar genes facilitates the subsequent steps in the proposed method, especially the steps involving feature selection. This is because the presence of similar genes, particularly those with high variance, affects the mean and median values for the expression of individual genes, thereby affecting the next filter steps. In a previous study [48], the ratio of the mean was used as a basis for removing similar genes. It has been proven that when there are outliers in the features, the application of the median criterion is a better choice than the application of the mean criterion since the mean values of gene expression are affected by the variance. However, the median is not a strong statistical criterion to depend on throughout the experiments, and it is not popular. Therefore, in this study, a new approach was taken in which genes whose expression showed very high variance were identified among the genes in each class. Since high variance leads to mean values that do not reliably present the population, it creates a non-desirable result with respect to feature selection. To avoid this problem and to facilitate the next steps in the analysis, in this study the mean and median ratios are applied in different contexts. The ratio of the mean values is used in the case of genes whose expression values do not show high variance within the class, whereas for genes with high variance within the class the ratio of the median values is applied. This strategy is used in a creative way to overcome the problems associated with the variance of the dataset. In this approach, the features in each class are divided into two groups according to their variance. A set of high-variance features (variance >15%) are separated from those with low variance (variance = <15%). The median ratio criterion is then applied to the high- variance group, and the mean ratio criterion is used for the rest. This is performed similarly for both classes of observations. The next step in the analysis reduces the high-dimensional features of the dataset by removing genes that have quite similar medians in both classes as well as those that have quite similar means in both classes. In this step, features for which the median and mean ratios for both classes are between 0.95 and 1/0.95 are removed from the dataset. This threshold range is chosen intentionally to remove the non-significant genes from the whole dataset as well as to reduce the effect of high-variance genes, hence making the next steps of the analysis smoother. By following this procedure, the number of genes in the dataset is significantly reduced.

### Selection using statistical filters

The reduced set of genes identified in the previous steps is used as input for three gene selection methods that are based on the filter approach. The statistical filters are the two-sample t-test (TT), feature correlation with class (COR) and the Wilcoxon rank sum test (WRS). Each of these depends on a specific statistical criterion for feature selection. The reason for choosing more than one filter is that, because the methods have different relative power, the use of a combination of these methods might yield better selection performance than the use of a single filter [32, 48]. Prior to the application of the filters, the dataset was divided into two parts; one part consisted of 85% of the data and was used in the process of model training and validation (testing); the other part, which consisted of 15% of the data, was set apart as a non-involved set to be used as a new real-world dataset for gene classification based on the predefined model. This was done because researchers in the field of data science have recently recommended that the whole process should be divided into three main steps, namely, training, validation and testing [49]. The best approach is to apply training and 10-fold validation to avoid overfitting. Hence, the last step in the process would involve generalizing the model against new datasets that may be obtained in the future. Since in our study no new separate dataset was available, we set aside a portion of the data to be used at the last step of the analysis as a new real-world case study. Moreover, utilization of the whole dataset for feature selection produces a biased result that does not demonstrate the real ability of the model during the test phase. Therefore, the statistical filtering was repeated in 10-fold runs on the trained dataset. In each method, the positions of the filtered genes in all the runs were compared based on their position weights. Next, the weight values were summed, and the genes were ordered from most attributed to least attributed according to the final ranks achieved within the 10-fold runs. The equation used in this calculation is a global weight equation that is given by

$$w(f)\;=\;\sum_{i\;=\;1}^{K}w_{i}(f)$$ (1)

where each *i* in *K* = the number of current fold iterations in the whole 10-fold run.

The first applied filter was the t-test, which is a univariate filter feature selection that is often employed in binary class applications [50, 51]. The common assumption of the t-test is that the values for the two compared groups of genes are normally distributed. The null hypothesis of the t-test assumes equal means and equal variances, and the alternative hypothesis rejects this assumption. The equation of the t-test is

$$t\;=\;\frac{c_{1}-c_{2}}{\sqrt{\frac{{\sigma_{1}}^{2}}{n}+\frac{{\sigma_{2}}^{2}}{m}}}$$ (2)

where *n* and *m* denote the population sizes of the first and second classes, respectively. The result of the assessment calls *t*, which is equal to 1 or 0; 1 represents the rejection of the null hypothesis at the 5% significance level and 0 denotes the acceptance of the null hypothesis at the same significance level. The p-value is also returned by the test; a small value of *p* indicates a significant difference among the compared samples. For the autism dataset, a normal distribution of the expressed genes is not guaranteed due to the presence of outliers. Therefore, the non-parametric version of the t-test was considered in MATLAB programming by assuming unequal variances in the two classes. This method was adopted to provide a more accurate measurement. The t-test has long been used in the application of microarray feature selection [50]. It has powerful scalability when the number of features is high [51]. Some studies used filters such as the t-test as the only feature selection step followed by direct application of the classification algorithms [52, 53]. In the current work, the t-test is used as a filter followed by wrapper-based gene selection; after this, the classification algorithm is applied.

The second applied filter method was feature correlation with class (COR), a univariate filter feature selection method that can be used as a pre-selection step in microarray gene selection [54, 55]. The value of feature discrimination, *S(f)*, is expressed by

$$S\left(f\right)=\frac{\sum_{k=1}^{K}P_{k}{\left(c_{k}-c\right)}^{2}}{\sigma^{2}\left(f\right)\sum_{k=1}^{K}P_{k}\left(1-P_{k}\right)}$$ (3)

where *c* is the mean value for the gene among both classes, *c*_*k*_ is the mean value for the *k*^*th*^ class gene, *σ*^2^(*f*) is the gene variance, and *P*_*k*_ is the probability of appearance of the *k*^*th*^ class in the dataset. A high value of *S(f)* represents good discrimination capability of feature *f* in distinguishing a particular class from other K classes. Here, the number of classes is two, so K = 2.

The third applied filter method was the Wilcoxon rank sum (WRS) test. Because the WRS test is a non-parametric filter method [56], it is not necessary for the gene expression data in the classes to be normally distributed. Hence, at first glance, it appears more appropriate to apply the WRS test to the present dataset. The rank sum test is also known as the Mann-Whitney test [57, 58]. To distinguish between the two classes, the criterion used by this test is based on the median value. The test compares the medians of the samples and produces the result as a ranking instead of as numerical values [59]. By arranging the results in ascending order, the rank and the index value of the arrangement are determined. The WRS test considers as the null hypothesis the hypothesis that all genes originate from one class. The statistical formula of the Wilcoxon rank sum is as follows [60]:

$$s(g)\;=\;\sum_{i\in N_{0}}\sum_{j\in N_{1}}I((x_{j}^{\left(g\right)}-x_{i}^{\left(g\right)}))\leq 0$$ (4)

where *I* is the function used to distinguish the classes. If the logical expression $(x_{j}^{\left(g\right)}-x_{i}^{\left(g\right)})\leq 0$ is true, *I* is 1; otherwise, it is 0. $x_{i}^{\left(g\right)}$ is the expression value of gene *g* in sample *I*, *N*_*0*_ and *N*_*1*_ represent the number of observations in each of the two classes, respectively, and *s(g)* denotes the difference in the expression of the gene in the two classes. Based on whether *s(g)* becomes 0 or reaches the maximum of *N*_*0*_ *× N*_*1*_, the considered gene is ranked in importance in the classification process. The following equation is used to calculate the gene’s importance:

$$q(g)\;=\;\text{max}(s(g),\;N_{o}\times N_{1}-s(g))$$ (5)

This method was used in the literature for the pre-selection of genes [60, 61], and it was shown to produce a powerful statistical result, especially when the data are severely skewed and approximately symmetric [62]. Usually, at the end of the analysis, WRS will give the rank of the genes, beginning with the most discriminative genes and proceeding to the less discriminative ones.

### Selection using a wrapper-based GBPSO-SVM algorithm

The last step in the selection of discriminative genes was conducted using geometric binary particle swarm optimization (GBPSO) in wrapper form with the support vector machines (SVM) algorithm; in this method, the GBPSO uses the accuracy prediction of the SVM to choose the best subset of genes. GBPSO begins with a random number of selected genes and searches for the optimal subset of genes in each iteration. The SVM classifier is used to evaluate the performance of each candidate subset using 10-fold cross-validation. The GBPSO algorithm leads to the selection of an optimal subset of genes that provides the best classification accuracy. Furthermore, it chooses the most discriminative genes to contribute to the next generation of gene subsets. Thus, each new candidate subset of genes is usually better than the previous subset.

**Fig 2:**
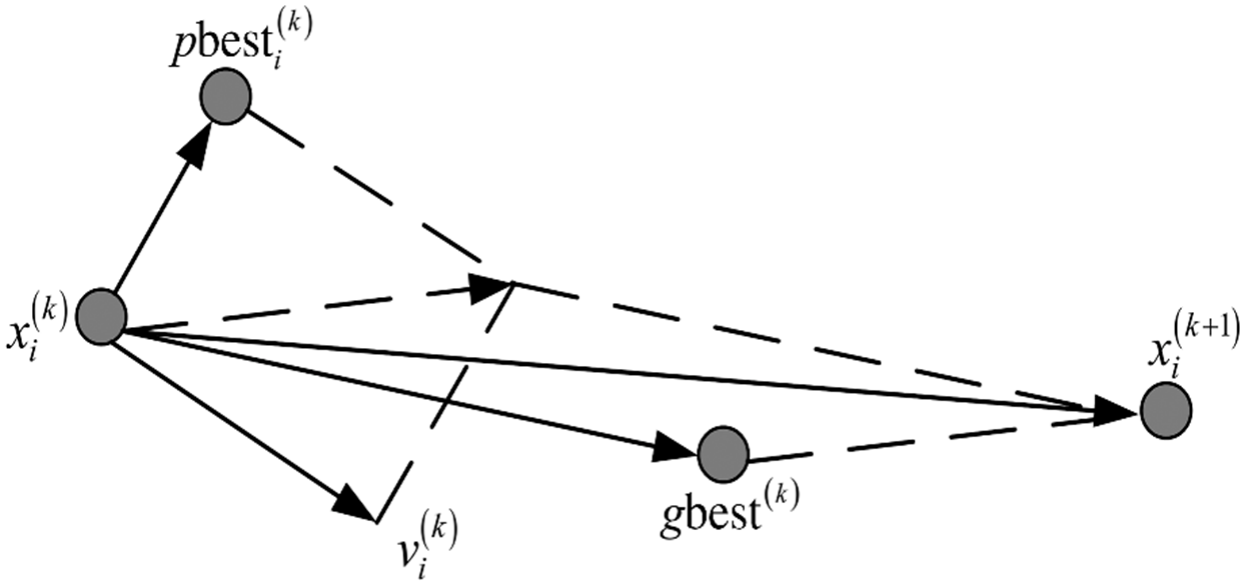
Illustration of the PSO principle.

Particle swarm optimization (PSO) is a stochastic population-based optimization technique that was first suggested by Kennedy and Eberhart (1995). PSO has received a great deal of attention from researchers in various fields due to the simplicity of its implementation and its rapid convergence towards acceptable solutions [35, 36, 63]. The PSO algorithm was inspired by the social behavior of birds flocking and fish schooling. The prototype algorithm of PSO comprises three steps: generating the positions and velocities of particles, updating their velocities, and finally updating their positions [37]. In PSO, a swarm is made up of individuals known as particles that communicate with each other through iterations to search for optimal solutions while they are moving in the search space [35]. Fig 2 shows the principle of particle movement in PSO. In each iteration, a particle velocity is updated according to the personal best (*p*_*best*_) and the global best (*g*_*best*_), where *p*_*best*_ is the best position that the particle has explored and *g*_*best*_ is the best position among all particles in the swarm. By assuming a search space having *D* dimensions, the *i*^th^ swarm particle can have a *D*-dimensional position vector represented by *X*_i_ = [*x*_*i*1_, *x*_*i*2_, …; *x*_*iD*_]. The velocity of the *i*^*th*^ particle is therefore denoted by *V*_i_ = [*v*_*i*1_, *v*_*i*2_, …; *v*_*iD*_]. It is also considered that the visited position that produces the best fitness value for the particle is *P*_*B*i_ = [*p*_*bi*1_, *p*_*bi*2_, …; *p*_*biD*_], while the best explored position so far is *G*_*B*_ *=* [*g*_*b*1_, *g*_*b*2_, …; *g*_*bD*_]. Thus, each particle’s velocity is updated based on the following equation:

$$v_{id}^{new}\;=\;w.v_{id}^{old}+c_{1}.{rand}_{1}(\ldots )\times ({p_{best}}_{id}^{old}-x_{id}^{old})+c_{2}.{rand}_{2}(\ldots )\times ({g_{best}}_{id}^{old}-x_{id}^{old})$$ (6)

where *d = 1*, *2*…, *D*, *c*_*1*_ is the cognitive learning factor and *c*_*2*_ is the social learning factor. The inertia weight (*w*) acts to reduce the particle’s velocity in steps and hence controls the swarms. The *w* value is usually between 0.4 and 0.9, whereas the random variables *rand*_*1*_ and *rand*_*2*_ have values that are uniformly distributed between 0 and 1 [35].

Consequently, the particles’ velocities are bounded within the range [*v*_*min*_, *v*_*max*_]. These bounds maintain the vector function of the velocity to avoid very abrupt movements of particles in the search space. The formula that is used to update the particle’s position is

$$x_{id}^{new}\;=\;x_{id}^{old}+v_{id}^{new}$$ (7)

where *d* = 1, 2, …. *D*, *i* = 1, 2, ….. *N*, and *N* is the size of the swarm.

Binary PSO (BPSO) is a modified version of standard PSO that was developed to handle variables with discrete design [37], whereas the original PSO was proposed for continuous variables. When BPSO is used for gene selection, a gene subset is expressed by a string vector of *n* binary bits *X*_*i*_ = (*x*_*1*_, *x*_*2*_,…*x*_*n*_) comprising ‘0’ and ‘1’. Consequently, if *x*_*id*_ is ‘0’, then the *d*^*th*^ gene is not selected in this subset, and an *x*_*id*_ of ‘1’ is alternatively chosen in the subset. In this regard, each binary string vector (*X*_*i*_) defines the particle’s position in BPSO. For instance, a particle with seven genes is encoded as ‘0100010’, implying that the second and sixth genes are selected. Therefore, the length of each particle is initially the same as the number of genes in the dataset.

The population of particles is randomly initialized. However, it is effective to initialize the particles in such a way as to produce better selection results. In the geometric version of BPSO, the particle’s current position, its *p*_*best*_ and its *g*_*best*_ are used as the three parents in a three-parent mask-based crossover operator (3PMBCX) to create a new position for the particle instead of using velocity. The equation for position updating is as follows [36, 43]:

$$x_{id}^{new}\;=\;{w_{1}.x}_{id}^{old}+w_{2}.{p_{best}}_{id}^{old}+w_{3}.{g_{best}}_{id}^{old}$$ (8)

where, for each element in the crossover mask, *w*_*1*_, *w*_*2*_ and *w*_*3*_ indicate the weight values associated with each parent represented by $x_{id}^{old},\;{p_{best}}_{id}^{old}$ and ${g_{best}}_{id}^{old}$, respectively. A condition is that the geometric crossover forces *w*_*1*_, *w*_*2*_ and *w*_*3*_ must be non-negative and must sum to one. In addition, an operator with a probability value of 0.01 is added to take care of bit-flip. This is to avoid early convergence. The advantage of this version of GBPSO is that it enables the generalization of PSO to virtually any solution representation in a natural and straightforward way [36]. The key issue of the GBPSO is the concept of particle movement. In this approach, instead of the notion of velocity added to the position, a three-parent mask-based crossover (3PMBCX) operator is applied to each particle to move it. According to the definition of 3PMBCX [43], given three parents a, b and c in {0, 1}^n^, a random crossover mask of length *n* with symbols from the alphabet {a, b, c} is generated. The offspring filling each element with the bit from the parent appearing in the crossover mask at the position is then built. The detailed parameters of the GBPSO model are illustrated in Table 1.

**Table 1:** Detailed parameters of the GBPSO model.

	Individual weight	Inertia weight	Social weight
**3PMBCX parameters**	0.34	0.33	0.33
**Mutation probability**	0.01		

In the current work, GBPSO is used as a wrapper feature selection method with a support vector machine (SVM). The support vector machine (SVM) algorithm is used because it is able to provide reasonable classification accuracy for high-dimensional data despite the availability of limited training samples.

Support vector machines are a group of supervised machine-learning methods known as a support vector network; they were developed by Vapnik [64]. The forms of this algorithm are widely applied in a variety of real-world problem domains [18, 36, 63], especially for gene classification of diseases [65–67]. Furthermore, the LIBSVM algorithm, which is a type of software for SVM classification and regression, was utilized by Liu et al. [68] for effective identification of human pre-microRNAs and hence to discriminate real pre-miRNAs from false ones. Moreover, SVM can perform both linear and nonlinear separable data classification. In the linear case, the boundary of linear decision is performed such that the smallest distance between the training samples and the boundary (margin) is maximized. The training data samples near the class boundary and along the hyperplanes are known as support vectors [18]. Nonlinear data can be handled by SVM upon mapping the gene space of low dimensionality extracted from the input space into a gene space of high dimensionality to achieve efficient classification. A cost is involved to consider the wrongly classified examples if there are linearly inseparable mapped data points, while the margin is maximized along with minimization of the cost [66]. Another property of SVM is that the number of coefficients to be determined is essentially dependent on the number of samples rather than on the number of genes. This is a useful characteristic of SVM for microarray data due to the presence of a low ratio of samples to genes in this type of dataset. However, it has been shown that decreasing the number of genes increases SVM performance [36, 69]. To utilize SVM as a classification algorithm in gene expression and sequence datasets, kernel functions are usually used. This allows the user to obtain an orthogonal hyperplane to distinguish the genes in a specific dimension. A number of research works have used SVM for gene selection or classification or both using different types of kernels [36, 70, 71]. This is because each type of kernel is suitable for different data. However, because it is not initially known which kernel is best for a specific set of data, it may be necessary to test multiple SVM types. Liu et al. [72] employed LIBSVM in a python package for DNA/RNA and protein/peptide sequence analysis based on pseudo components and kernel methods. In their studies, the kernel function of the radial basis function (RBF) was used to train the SVM classifier; in the current study, the polynomial kernel is applied owing to its higher classification accuracy for our dataset. Following further optimization of the kernel parameters, the identification of DNA-binding proteins by incorporating amino acid distance-pairs and reduced alphabet profile into the general pseudo amino acid composition upon a new predictor (iDNA-Prot|dis) outperformed the existing predictors for the same purpose [73]. Liu et al. also reported that each kernel contains different discriminative information and that combining the kernels automatically is, therefore, a promising way to improve the performance of the model. Consequently, the combination of sequence-based kernels with evolutionary information extracted from frequency profiles, in which three top-performing sequence-based kernels (SVM-Ngram, SVM-pairwise and SVM-LA) were combined with the profile-based protein representation, was proposed to predict protein remote homology [74]. In this study, SVM was applied using the polynomial kernel because this kernel method showed the highest classification accuracy.

**Fig 3:**
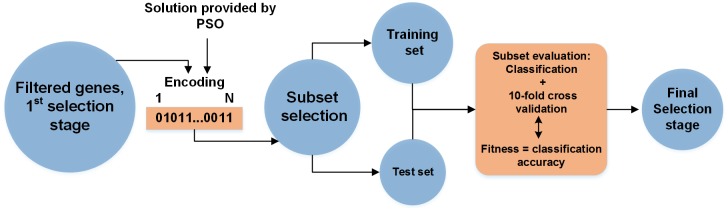
Application of GBPSO-SVM in gene selection.

The fitness function in GBPSO is used as an evaluator to select the best subsets of features, which are constructed based on the accuracy so far obtained by the SVM classifier. The particles having the best fitness values are recorded to maintain the optimal solution for a given population. This defines the best subset of genes and gives better accuracy. This is applied in 10-fold cross-validation such that the entire training set can be used in the process of finding the best genes. Fig 3 shows the operation principle of the GBPSO-SVM method, in which the genes are expressed from the dataset and the best subset of genes is selected. The PSO particles are represented by vectors of bits, where each bit corresponds to a specific gene. A gene is retained in the subset if it holds an encode value of 1 and is not included in the subset if it holds an encode value of 0. Hence, the number of genes in the dataset determines the length of each particle.

## Results and discussion

### Dataset reduction

**Fig 4:**
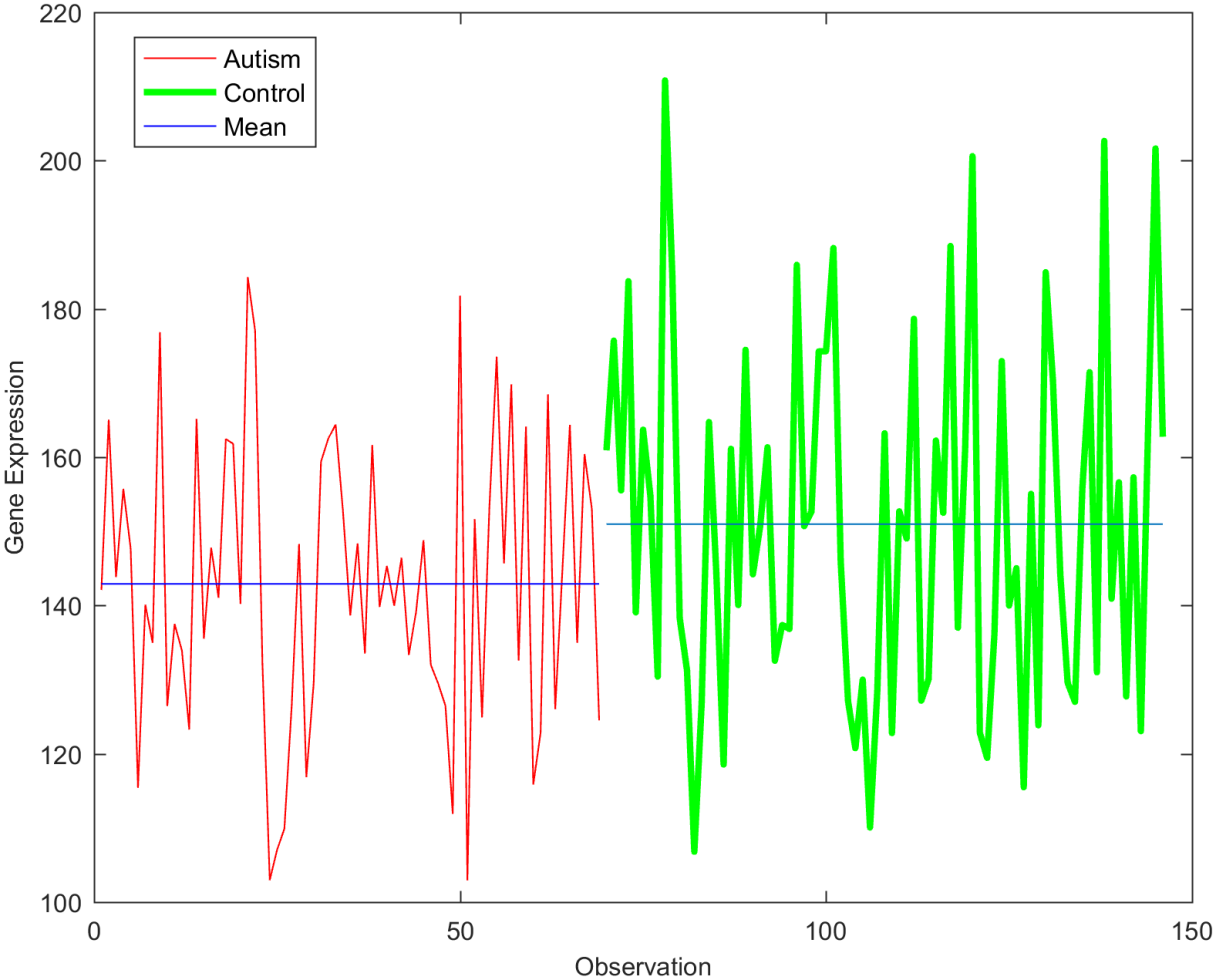
Expression of a representative gene in samples from control and autistic individuals.

Despite the presence of a high-dimensional dataset from autistic individuals, most of the features appeared as outliers, indicating that the gene expression values in the observations are highly varied. It was noted that the gene expression values of both classes exhibited a high variance, as shown in Fig 4. The deviation of the expression value of a gene from its mean value is statistically explained in terms of variance. It is certainly true that not every single gene is responsible for autism disorder or can be used as a discriminative biomarker. Therefore, to identify the attributed genes, the genes with similar mean values in the two datasets, i.e., those with mean ratios close to unity, should be removed. However, this approach does not yield an accurate result if it is directly applied to the current dataset. This is because several genes show high variance among both classes. Consequently, the utilization of the mean ratio criterion alone to remove the genes unrelated to autism from the two classes does not provide a reliable result. It was reported that due to the high variance in the expression of genes related to autism, the median ratio can be considered as an alternative to the mean ratio for removing similar genes [70]. Again, using the median ratio only is not a reliable approach since genes with low variance will be negatively affected under this reduction process.

**Fig 5:**
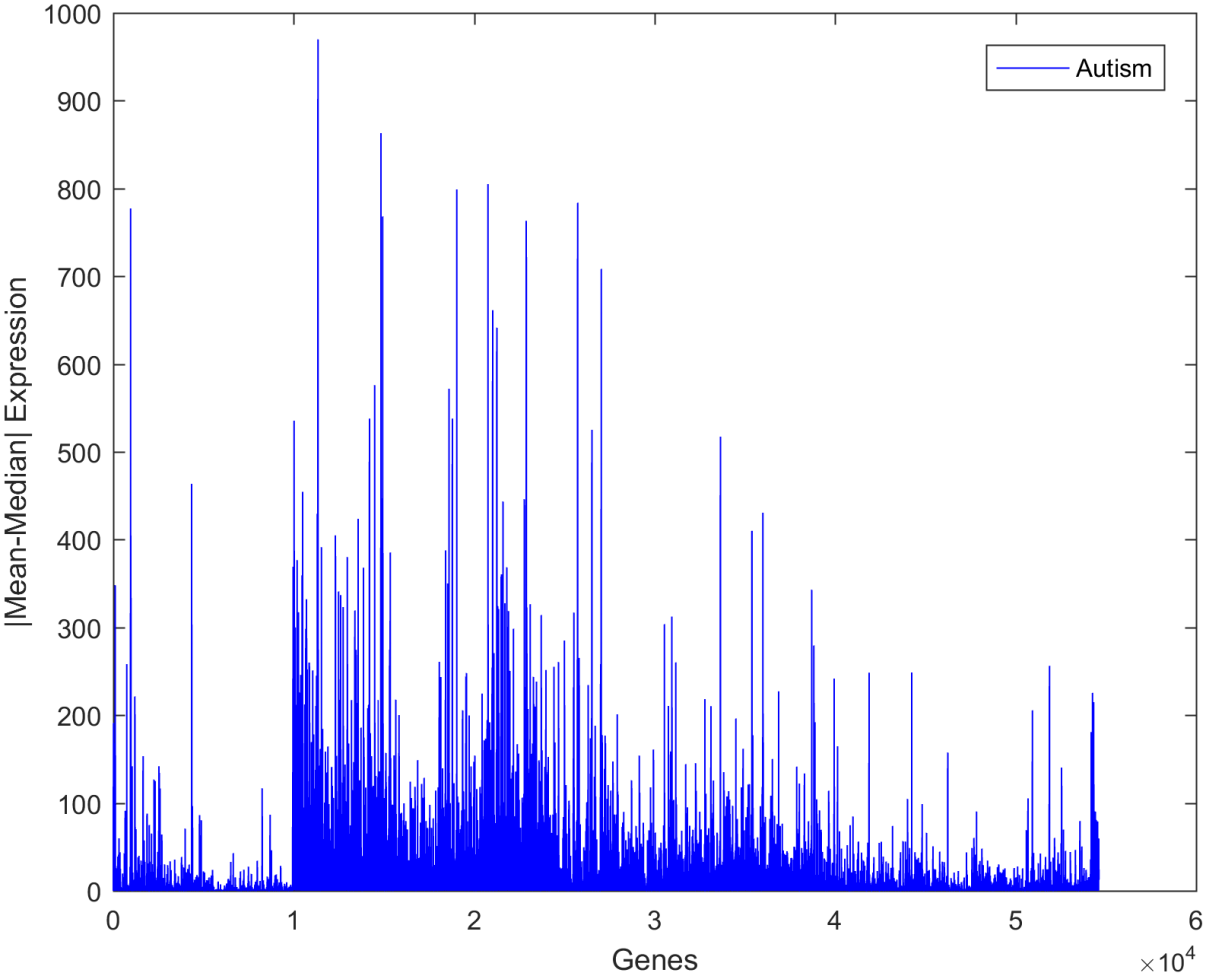
Absolute values of the differences between the mean and median values of gene expression for the autism observations.

**Fig 6:**
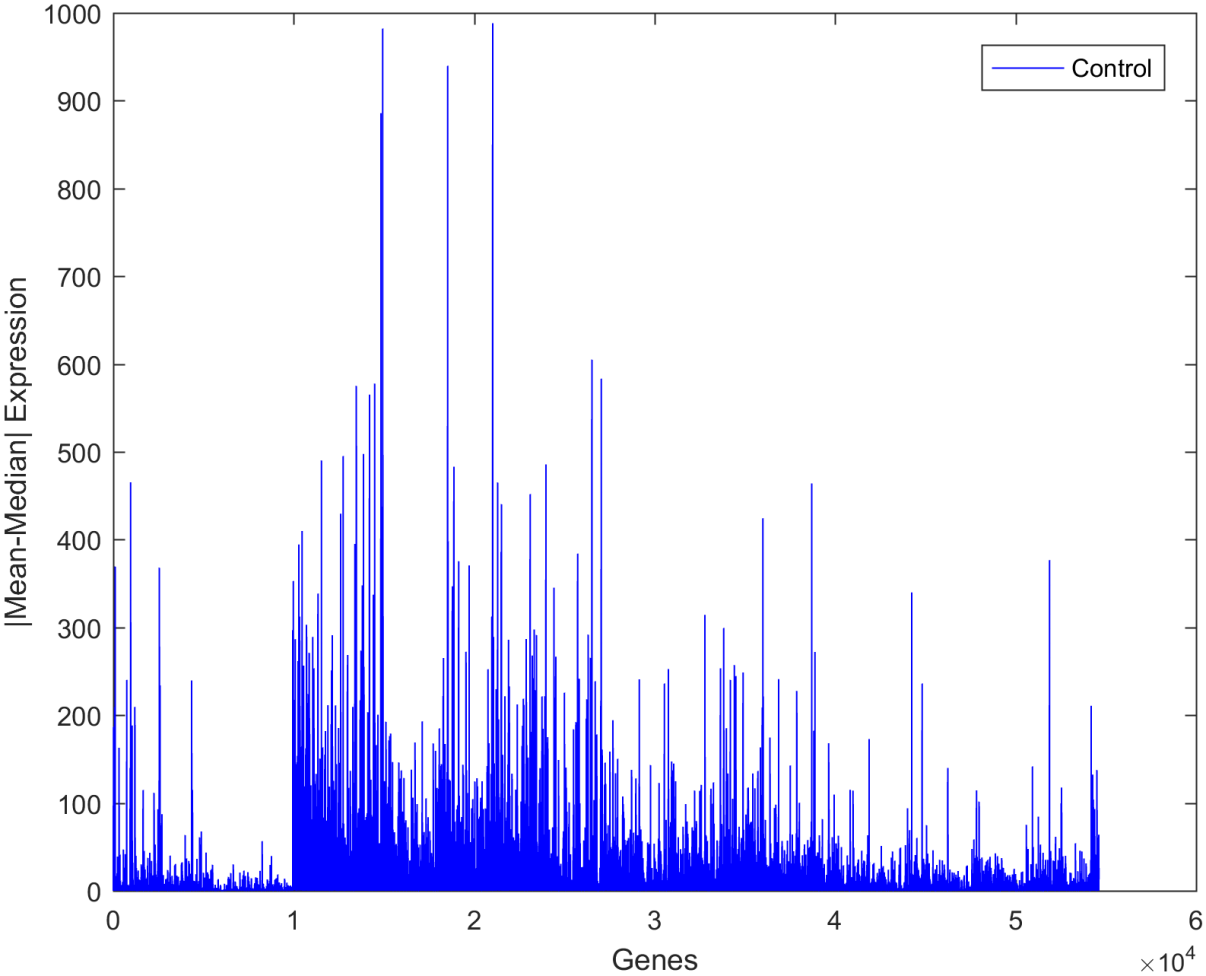
Absolute values of the differences between the mean and median values of gene expression for the control observations.

The absolute value of the difference between the mean and the median of the expression of individual genes was measured to determine the strength of outliers, as shown in Figs 5 and 6. It was seen that this variation is larger in the autism group than in the control group, implying that there are some alterations in the gene expression values in the autistic group. Upon close inspection of the figures, it is clear that this variance is not associated with only one gene or with a small group of genes but with a wide range of genes. This can be regarded as another confirmation of the fact that autism is a spectrum disorder and that several genes may contribute to the occurrence of the disorder.

**Fig 7:**
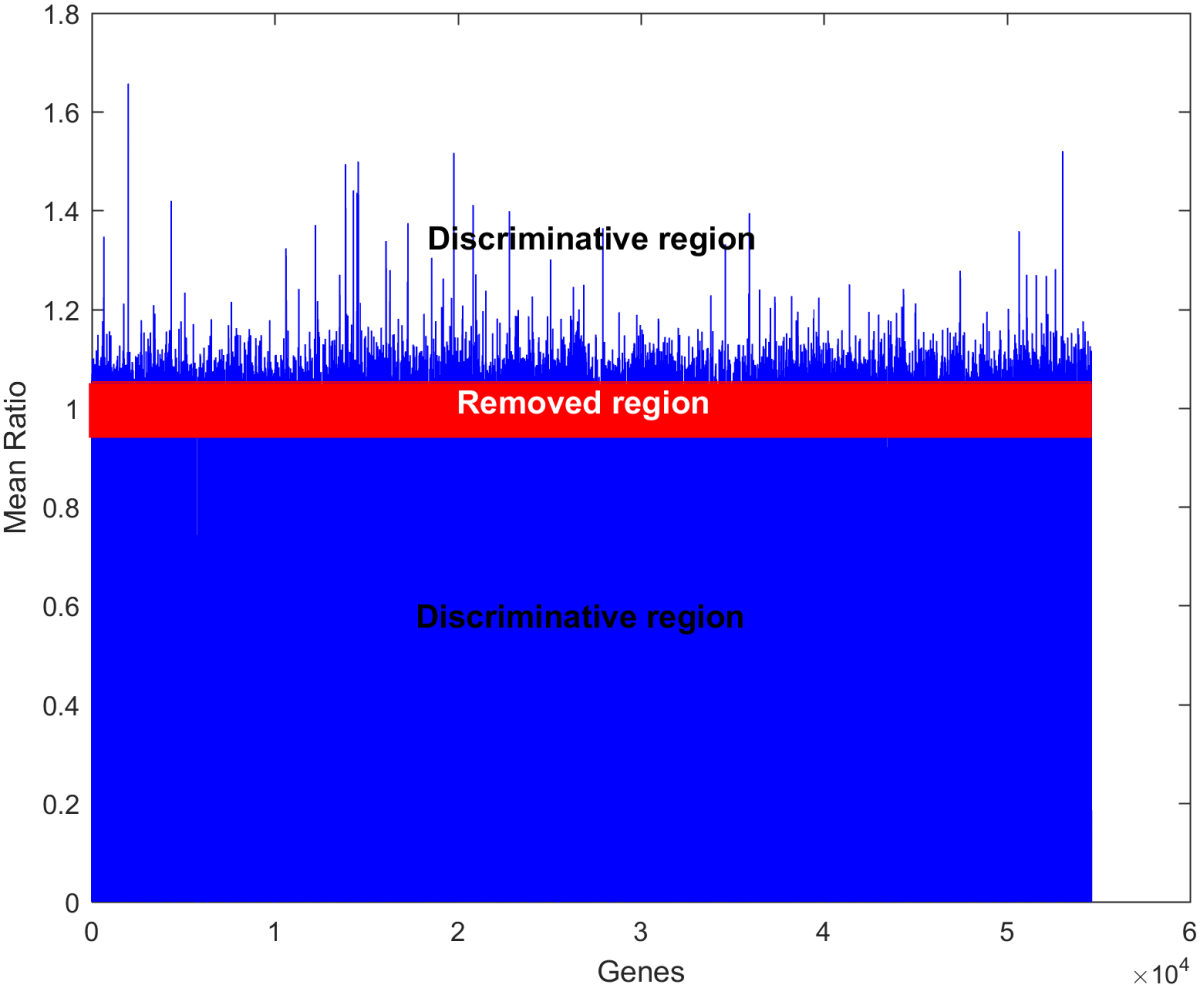
Illustration of the discriminative regions and removal of similarly expressed genes from both classes of observations.

It is seen that genes with high variance are more tolerated when the median ratio is applied, and vice versa. Therefore, to remove the most similar genes and to reduce the dataset, an alternative strategy was followed in this work. This strategy relied on the fact that genes with high variance in expression can be treated according to the median ratio criterion, whereas those with low variance can be treated according to the mean ratio criterion. In this way, among the genes that present variance of 15% and higher, the median ratio was applied to remove similar genes, and for genes with a variance of 15% or smaller the mean ratio was applied. Hence, genes with median ratios or mean ratios of 0.95 or greater were removed from both classes, as illustrated in Fig 7. Based on this removal process, the number of genes was reduced from 54,613 to 9454. The reduced dataset that was obtained at this stage is provided in the supplementary information as the S1 Dataset. It will be shown later that this reduction process improves the accuracy of the SVM classifier at threshold ratio of 0.95. In other studies [48, 70], a mean or median ratio threshold of 0.96 was used separately to reduce the dataset to 17,831 or 16,230 genes. In yet another study, a mean ratio of 0.98 was applied as a threshold to reduce the dataset to 16,230 [32]. In comparison, our reduced dataset of 9454 genes could help improve classification accuracy and reduce memory complexity.

### First stage of selection

The reduced dataset resulting from the reduction step is not satisfactory for direct application in the classification process or as a basis for building the model since, with 9454 genes, its dimensionality is still high, and not all of the genes are discriminant. Hence, further reduction is conducted using the three filtering methods TT, COR and WRS in parallel to select the most discriminative genes. It was observed that each filtering method identifies different sets of genes with a specific repetition of the discriminative genes among them (see S2–S4 Datasets). This is due to the high variance of several genes in the samples from individuals with autism disorder, indicating the necessity of using further selection steps. In this stage, the 200 most discriminative genes were selected based on their rank positions in descending order. Table 2 shows the percentage of similar genes that were selected by each method. The greatest similarity (93%) between the sets of 200 selected genes occurred between TT and COR, whereas WRS showed 69% similar genes with both TT and COR. It is noteworthy that the repetitive genes that appeared in the three filtered sets were not assigned the same ranking positions. The three discriminative genes that were assigned the highest rankings among 30 sets of filtered genes, i.e., within a 10-fold run for each filter, were ZSCAN18, CFC1B and CAPS2.

**Table 2:** Similarity percentages for sets of 200 discriminative genes selected by various filtering methods.

Filtering method	TT	COR	WRS
**TT**	100	93	69
**COR**	93	100	69
**WRS**	69	69	100

**Fig 8:**
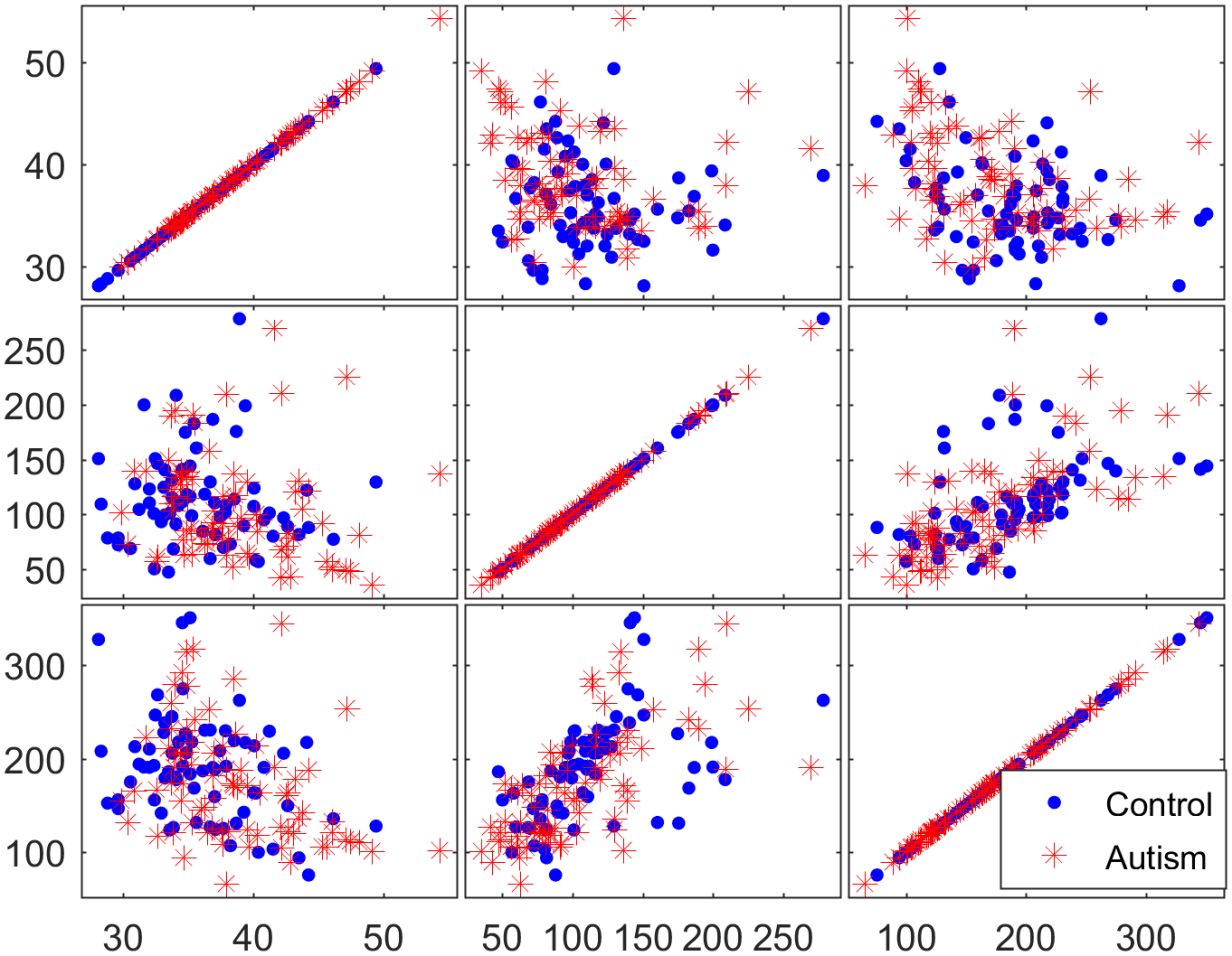
Matrix plots for three representative selected genes from the reduced dataset before the application of filter methods.

**Fig 9:**
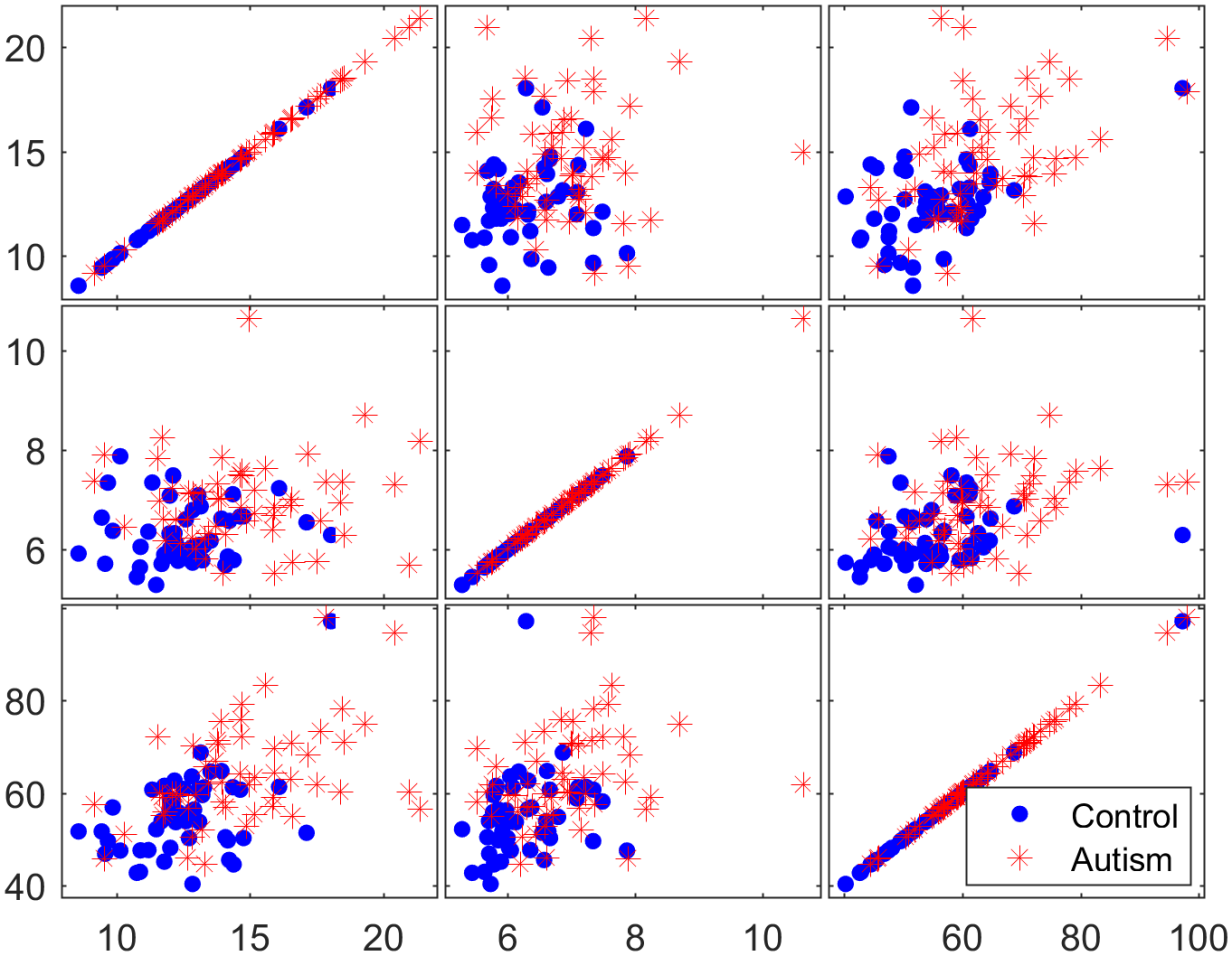
Matrix plots for three representative selected genes from the 200 genes filtered by the TT method.

**Fig 10:**
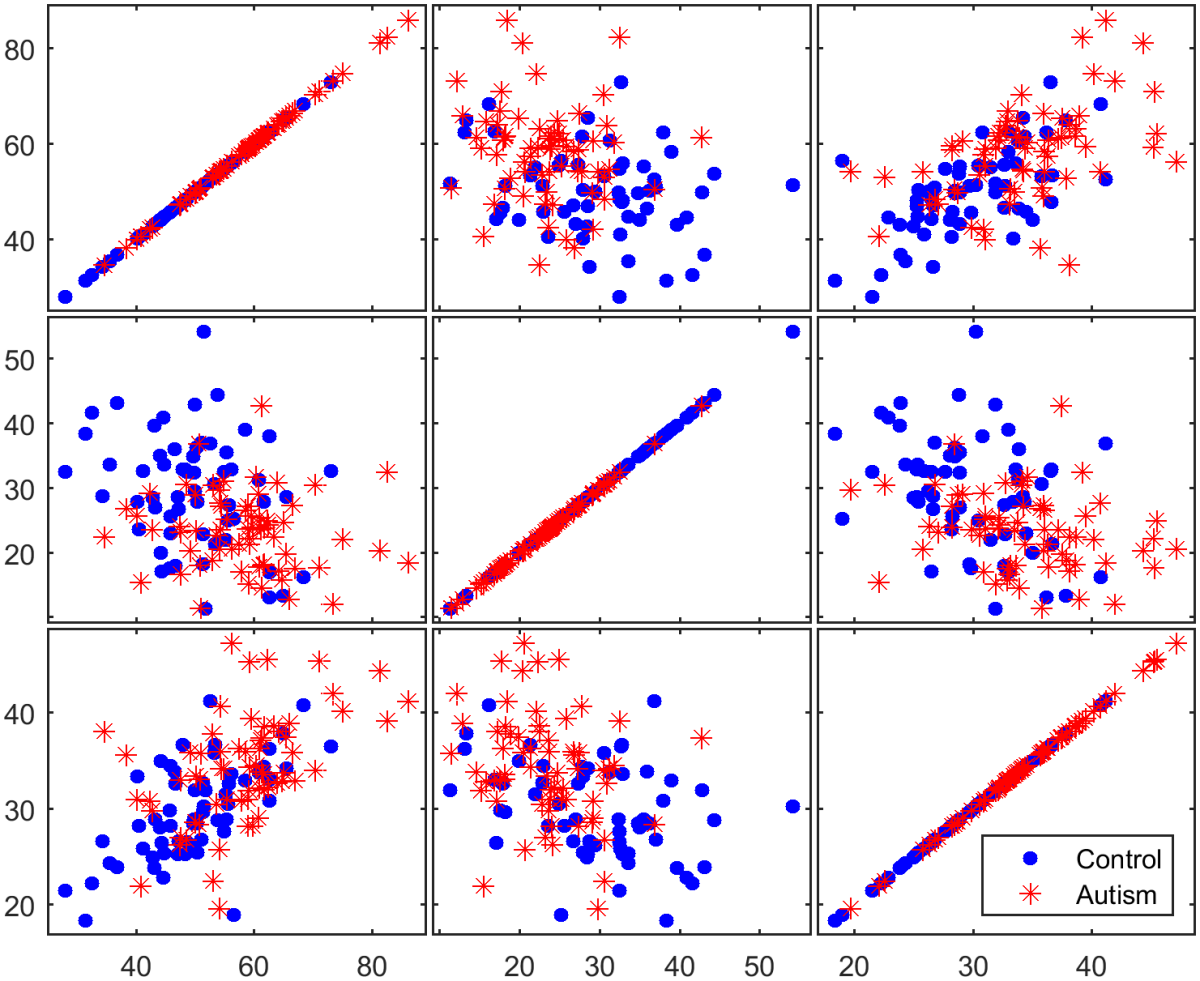
Matrix plots for three representative selected genes from the 200 genes filtered by the COR method.

To determine the discriminative ability of the genes identified in the filtration process, three selected genes were examined using matrix scatter plots. Figs 8–10 show a qualitative assessment of the genes obtained from the initial reduced dataset (before using filters) and of the genes obtained using the TT and COR filters, respectively. The significant impact of the filtration process on the selection of attributed genes, in which the expression values of the autism-related genes are clustered apart from those of the non-autism-related genes, is readily apparent in Figs 9 and 10. However, no such clustering occurs in the matrix plot of the genes extracted from the non-filtered dataset; in that plot, the expressed genes are uniformly distributed in the whole space without any pronounced clustering between the two classes of observation.

**Fig 11:**
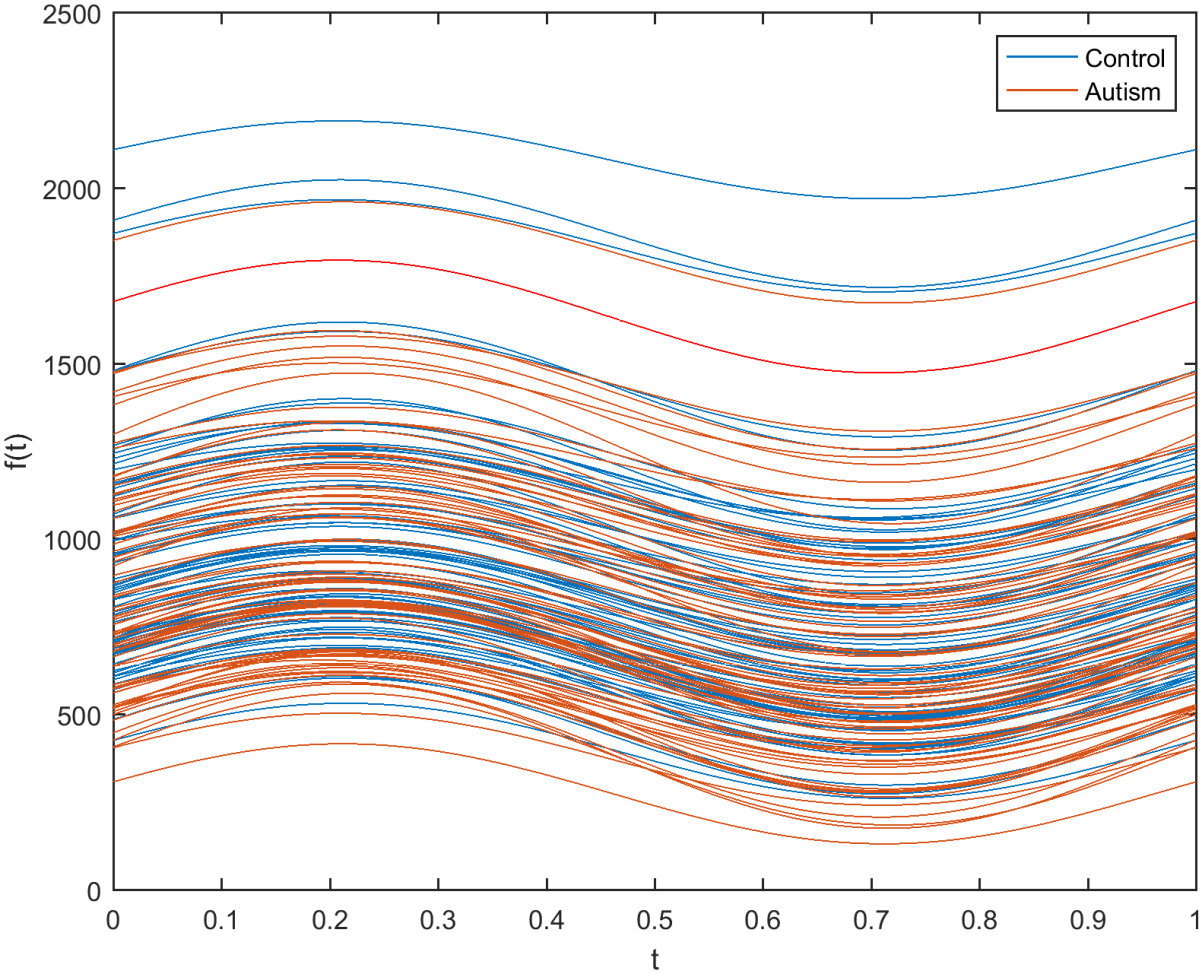
Andrews plot for three representative selected genes from the reduced dataset before the application of filter methods.

**Fig 12:**
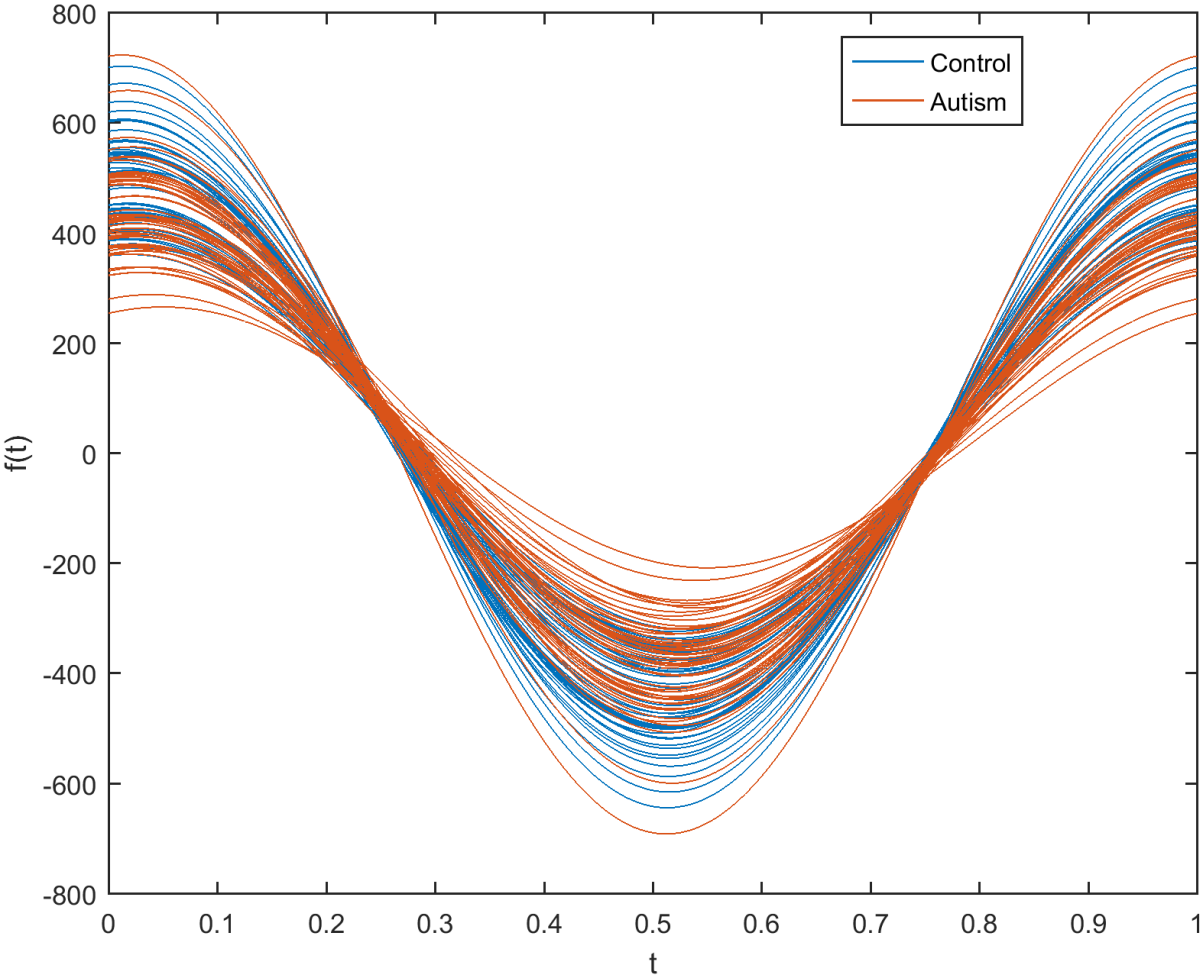
Andrews plot for three representative selected genes from the 200 genes filtered by the WRS method.

Another method of estimating the impact of filtration on the selection of discriminative genes in autism disorder used an Andrews plot. Figs 11 and 12 show Andrews plots for three genes selected from the initial reduced dataset (before using filters) and the dataset obtained using the WRS filter, respectively. In Fig 11, the gene expression for the non-filtered dataset appears as a wide bundle with no distinguishable separation between the two classes. It is worth noting that the filtration process narrowed and aggregated the expression bundle of the filtered genes, as shown in Fig 12, in which the autistic class of genes is separable from the non-autistic class.

### Classifier assignment

One of the most important tasks in conducting gene expression analysis using machine-learning algorithms is the building of a classification model that recognizes the discriminative genes with the highest possible accuracy. However, not every classifier works effectively on all datasets. For each dataset, a unique classifier or a limited number of classifiers typically work best. To explore this, the discriminative genes identified using different filtration methods were analyzed using six different classifiers, and the performance of each classifier was noted. Table 3 shows the accuracy of each classifier; of the tested classifiers, SVM had the highest accuracy.

**Table 3:** Accuracy percentages of six different classifiers used after the first selection stage in 10-fold cross-validation.

Classifier	TT filter	COR filter	WRS filter
Decision Tree	62.9	65.3	57.3
Discriminant Analysis	75.0	72.6	74.2
Logistic Regression	46.8	41.9	52.4
SVM	**86.3**	**81.8**	**83.8**
K-Nearest Neighbor	73.4	74.2	76.6
Ensemble Bagged Trees	71.0	69.4	69.4

Based on the classification results, SVM was chosen as the ultimate classifier to be combined with the GBPSO algorithm in wrapper form to perform the final stage of the gene selection and classification process, as will be discussed later. Further analysis was also conducted to elucidate the impact of dataset reduction on the accuracy of the SVM classifier. The details of this analysis are provided in Table 4. It was observed that the utilization of a combined mean and median ratio as a reduction criterion to remove the most similar genes results in remarkable improvement in the classifier accuracy. Furthermore, this combination was found to outperform the results obtained using the mean ratio or the median ratio alone. Using this method, the initial selection results showed better classification accuracy and less computational complexity, and the dimensionality of the dataset was reduced from 54,613 genes to 9454 genes. As such, the best classification accuracy of the SVM for the TT filtered genes in this stage was found to be 86.3%, higher than the values previously reported (of about 86.1% and 78.4%) for fused genes of eight filter methods [32, 48]. The improvement in the accuracy of the SVM classifier is attributed to the impact of the threshold value of mean and median ratio that is defined and used to remove the most similar genes.

**Table 4:** Accuracy percentage of the SVM classifier at different stages of removal of similar genes and applied filtration results for 200 discriminative genes.

Reduction criterion	Genes, #	TT filter	COR filter	WRS filter
Without reduction	54,613	72.7	75.0	68.2
Mean & Median ratio 0.99	37,125	72.7	75.0	68.2
Mean & Median ratio 0.98	26,644	72.7	75.0	68.2
Mean & Median ratio 0.97	18,975	75.0	75.0	70.5
Mean & Median ratio 0.96	13,470	77.3	75.0	77.7
Mean & Median ratio 0.95	**9454**	**86.3**	**81.8**	**83.8**
Mean & Median ratio 0.94	6655	80.4	78.5	82.5
Mean & Median ratio 0.93	4606	76.4	75.4	75.4
Mean & Median ratio 0.92	3231	73.5	74.5	73.5
Mean & Median ratio 0.91	2165	69.6	70.5	70.6
Mean & Median ratio 0.90	1490	72.5	69.6	71.5
Mean ratio 0.95	13,324	75.0	77.3	72.7
Median ratio 0.95	14,440	77.3	77.3	75.0

### Final stage of selection and classification

The last step of gene selection was conducted using the GBPSO optimization algorithm wrapped with the SVM classifier; in this step, those particles (gene subsets) having the best values of fitness were recorded to maintain a better solution at given population. As such, the best subset of genes that provided the highest classification accuracy was identified and returned. Table 5 shows the classification accuracy (10-fold cross-validation) as well as the prediction accuracy for the new dataset using the model. Gene subsets #1, #2 and #3 correspond to the sets of discriminative genes that were identified using the TT, COR and WRS filters, respectively (see S5–S7 Datasets). It is worth noting that the merged form of these subsets (the S8 Dataset) produced the highest classification accuracy of 92.1%, which is higher than previously reported classification accuracies [32, 48, 70]. The inclusion of the genes in each GBPSO-SVM branch and in the fused set was based on the number of times that gene was repeated in the 10-fold selection. Genes with repeatability of less than 7-fold were not included in the sets. The improvement in classification accuracy may be due to the effect of stepwise selection procedures that were followed during the pre-selection operations as well as the incorporation of a relatively high number of filtered genes (200 genes) at the final stage of selection by the GBPSO-SVM algorithm. In previous studies [75], superior performance of PSO over GA in terms of accuracy was reported. To determine the real difference between the time taken by GA and GBPSO, in this study GA was tested against GBPSO. It was observed that the time required for feature selection by the GBPSO method is one-third of that required by the GA method. Moreover, GBPSO involves fewer steps and requires less memory to perform feature selection. The ten most frequent repetitive and/or similar genes in the 10-fold selection among the three achieved subsets were FKBP4, RHPN2, SEMA6B, ZNF230, LARS, LOC283075, CAPS2, ANKUB1, B3GNT7, and CASP2. A comparison between the most discriminative genes that were chosen during the initial and last selection steps identified a common gene, CAPS2. Therefore, it might be possible for us to assign this gene as one of the most important ASD risk genes. It was reported that the Ca^2+^-dependent activator protein for the CAPS family of secretory proteins regulates neuropeptide-containing dense-core vesicles (DCVs) at sites of secretion such as nerve terminals [76]. It was also claimed that genes associated with autism are responsible for Ca^2+^ regulation in brain membranes, and CAPS2 is one of the genes that contribute to the regulation of Ca^2+^ levels [77]. Consistent with our observations, a recent study showed that CAPS2 may be a risk factor for autism [78].

**Table 5:** Accuracy percentage of the SVM classifier at the final stage of gene selection by the GBPSO-SVM algorithm in 10-fold cross-validation and the accuracy of the new dataset in terms of the model.

Dataset	Classification accuracy, %			
	Gene subset #1	Gene subset #2	Gene subset #3	Merged set
Validation/testing set	91.1	89.5	87.3	**92.1**
Non-involved set	83.2	80.1	82.5	84.7

## Conclusions

The gene expression values of autism spectrum disorder (ASD) were successfully analyzed with the goal of improving the selection and classification process. This was accomplished using a combination of statistical filters and a wrapper-based GBPSO-SVM algorithm. It was noted that the expression of genes potentially associated with ASD varies greatly among the observations; hence, the utilization of the mean ratio criterion alone to remove similar genes does not provide a reliable result. Instead, both the mean and median ratio should be utilized simultaneously. It was shown that the pre-reduction process improves the accuracy of the SVM classifier. The results showed that each filter method identifies different sets of genes with a specific repetition of the discriminative genes among them. This is due to the high variance of several genes in autism disorder and necessitates the use of additional selection steps. During the filtration stage, the three most discriminative genes that received the highest repetition ranking among 30 sets of filtered genes were found to be ZSCAN18, CFC1B and CAPS2, whereas after further gene selection using GBPSO-SVM, a set of ten genes, namely FKBP4, RHPN2, SEMA6B, ZNF230, LARS, LOC283075, CAPS2, ANKUB1, B3GNT7, and CASP2, was selected. A comparison of the most discriminative genes identified during the initial and final selection steps pointed to the existence of a common gene (CAPS2), which was designated as the gene that showed the greatest association with ASD risk. The merged forms of the gene subsets that were selected by the GBPSO-SVM wrapper produced an improved classification accuracy of 92.1%, higher than those reported previously, in spite of its improved efficiency. This enhancement was attributed to the effect of using GBPSO-SVM as an accurate and fast algorithm.

## Supporting information

S1 Dataset The reduced dataset. The dataset contains 146 observations and 9454 genes.
(CSV)

S2 Dataset The dataset selected by the TT filter. This dataset contains 124 observations and 200 genes.
(CSV)

S3 Dataset The dataset selected by the COR filter. This dataset contains 124 observations and 200 genes.
(CSV)

S4 Dataset The dataset selected by the WRS filter. This dataset contains 124 observations and 200 genes.
(CSV)

S5 Dataset The first dataset selected by GBPSO-SVM. This dataset was derived from the dataset selected by the TT filter. It contains 124 observations and 48 genes.
(CSV)

S6 Dataset The second dataset selected by GBPSO-SVM. This dataset was derived from the dataset selected by the COR filter. It contains 124 observations and 46 genes.
(CSV)

S7 Dataset The third dataset selected by GBPSO-SVM. This dataset was derived from the dataset selected by the WRS filter. It contains 124 observations and 37 genes.
(CSV)

S8 Dataset The merged set of the datasets selected by GBPSO-SVM. The merged set was generated by fusing the three datasets selected by GBPSO-SVM. It contains 124 observations and 101 genes.
(CSV)
